# Experimental Assessment of Multiple Properties of Mycelium-Based Composites with Sewage Sludge and Bagasse

**DOI:** 10.3390/ma18061225

**Published:** 2025-03-10

**Authors:** Min Hu, Xuejuan Cao

**Affiliations:** 1 School of Civil Engineering, Chongqing Jiaotong University, Chongqing 400074, China; smilydream@scac.edu.cn; 2 Sichuan Engineering Research Center of Urban Sludge for Building Materials Resource Utilization, Sichuan College of Architecture &Technology, Deyang 618000, China

**Keywords:** low carbon footprint, sewage sludge, mycelium, biological compatibility

## Abstract

Mycelium-based composites (MBCs) have a lot of potential as an alternative lightweight material due to their small environmental footprint and their biodegradability. The unique properties of cellulose-rich sewage sludge (SS) allow it to be used as a substrate for manufacturing MBCs. In order to examine the feasibility of creating MBCs using SS, this study used SS and bagasse as nutrient substrates and cultivated MBCs on ready-made mycelium (*Pleurotus ostreatus*). The physico-mechanical properties, morphological properties, and thermal stability of MBCs were tested and analyzed. The results show that both the bagasse and SS promoted fungal growth to create a dense mycelial network on day 10. Adding SS increased the density and compressive strength. The volume shrinkage of the MBCs first decreased and then increased. The optimal ratio of ready-made mycelium–sewage sludge was 2:1. The thermal conductivity of the bagasse-based MBCs was 0.12 Wm^−1^K^−1^ and that of the SS-based MBCs was 0.13 Wm^−1^K^−1^. These physico-mechanical characteristics satisfy the requirements of lightweight backfill materials for use in highways. Additionally, the SS supported more robust growth of hyphae and resulted in stronger MBCs. In comparison to bagasse, it also showed better thermal stability and a higher residual mass. It is feasible to produce MBCs with SS, and the biocomposite proposed in this study could be used as a lightweight backfill material of the type that is widely needed for use in highway construction and maintenance.

## 1. Introduction

In recent years, mycelium-based composites (MBCs) have drawn the attention of an increasing number of researchers due to their low energy consumption and carbon emissions [1,2]. MBCs consist of fungi and lignocellulosic substrate materials; primarily via enzymes, the fungi degrade lignocellulosic substrate into nutrients, resulting in branching of hyphae. These branching filamentous mycelium interface with the dispersed lignocellulosic substrate through a three-dimensional interconnected network, serving as a natural binder. Unlike conventional manufacturing processes, the mycelial branching process occurs naturally; self-growth manufactures the resulting MBCs as needed when the colonization conditions are optimized [3]. They consume less energy [1], emit less carbon [4], and can be used as biofoam [5,6,7,8,9] for packaging [10] and construction [11,12] and as impact-resistant filling materials [13], including for design purposes [14]. Agricultural by-products may include lignocellulosic components, and a few studies have investigated agricultural by-products comprising cellulose, hemicellulose, lignin, and other polysaccharides; the cellulose in these ranged from 26 to 82.7%, while the hemicellulose was in the range of 0.15–33% [1]. Cotton has the highest amount of cellulose, at 82.7%, while hemicellulose in coconut husk was present at only 0.15%, along with a lignin content of up to 45% [1]. According to the literature, these agricultural by-products have been widely used as substrates to promote successful mycelial growth. César, E. et al. produced MBCs using rice husk, bagasse, coconut husk, and juncao grass. Visual inspection revealed that the composites achieved 70 to 80% mycelial colonization rates. Furthermore, juncao grass containing 2% lime and 10% wheat bran grew the fastest overall, with over 80% mycelium colonization, generating a high compressive strength of 78.34 kpa [15]. Canda, K et al. [9] added starch and gypsum to cotton plant biomass of different particle sizes to obtain MBCs, and the compressive strength was 72 kPa. A comparison of bran–cotton and bran–hemp mixtures showed that bran offered desirable nutrients capable of increasing mycelial growth. In addition, bran improved the mechanical properties of MBCs because bran particles synergistically increased composite strength and hardness via the filling effect and strengthening effect caused by reducing the voids [16]. To expand the sources of substrates, Sun et al. [17] cultured Trametes versicolor mycelium on yellow birch wood particles, and the materials formed from mycelia-modified wood had better properties than those made from physically mixing pure wood particles and mycelium. Moreover, MBCs became stiffer when the substrate was harder to digest [18]. The use of a stiffer substrate or a strain with stiffer mycelia is likely to yield MBCs with much more favorable physico-mechanical properties [19]. The use of nutritional substrate as inocula increased the overall density [7]. These MBCs are not only cost effective, lightweight, and biodegradable but also exhibit reduced carbon emissions and excellent physico-mechanical properties. 

Considering the characteristics of these MBCs, they have the potential to be used as lightweight backfill materials (LBMs). LBMs normally consist of soil, cementing material (usually cement), water, and expanded polystyrene (EPS) or foam agents [20,21]. In recent decades, they have been widely applied in the backfilling of embankments, slope protection, backfilling behind retaining walls and bridge abutments, and the backfilling of pipeline trenches [22]. However, the production of LBMs consumes large amounts of EPS or cement, which poses the environmental challenges of high energy consumption and carbon emissions [23]. A large body of research is devoted to green LBMs, with an emphasis on their low environmental impact and carbon footprint [24].

Sewage sludge (SS) is a semisolid by-product derived from municipal wastewater treatment. Owing to the rapid development of urbanization, the amount of SS is increasing, and its disposal has become an urgent issue. Some disposal methods for SS, such as landfilling and land use, have become obsolete due to secondary pollution, while drying and combustion are not the main priority, owing to high energy consumption [25]. SS consists of a heterogeneous mixture of organic and inorganic matter, in which the organic components are mainly carbohydrates (cellulose, hemicellulose, and lignin), protein, lipids, or fats, and it could be a potential raw material for MBCs [25]. Prior to this study, there have been no reports on MBCs produced from SS. In our recent study, SS was found to consist of 64.4% cellulose, 2.7% hemicellulose, and 30.7% lignin, while bagasse contained cellulose, hemicellulose, and lignin at 55.2%, 25.3%, and 16.8%, respectively [26]. Substrates with higher cellulose content provide sufficient nutrients, supporting more robust growth and potentially resulting in stronger MBCs [27]. Because agricultural by-products are seasonal, their output is limited by a constant need for substrates. By contrast, cellulose-rich SS is similar to agricultural by-products and could be beneficial for the manufacture of MBCs as a valuable resource, providing essential nutrients to enable mycelial growth. Although there is no literature confirming the effect of inorganic matter of SS (primarily SiO_2_, Al_2_O_3_, and CaO) on mycelium growth, it is reported that MBCs were successfully developed utilizing sawdust and glass fines as substrates [9]. The higher-content aggregate improved the mechanical properties [24]. Due to the inorganic matter of laterite particles, they could control the stiffness and compressive strength of the resulting MBCs [28]. The inorganic particles embedded in the mycelium network are able to provide mechanical support for the resulting MBCs [29]. It is also reported that fungi also readily spread on inorganic glass fines. However, because the high silica content limited fungal growth, about 50 wt% organic material was supplemented to facilitate sufficient growth [30]. Due to the heterogeneous mixture, SS is a promising alternative to agricultural by-products.

Thus, the aim of the study is to examine the potential of using SS as a substrate source bound together by fungal mycelium in designing MBCs, which can be used as a nutrient supplement in MBC production purposes. Specifically, the objectives are to develop an experimental protocol for culturing *Pleurotus ostreatus* in three different substrate types, namely bagasse (BM), a mixture of bagasse plus sewage sludge (BSM), and sewage sludge (SM) to prepare the desired MBCs, and then to investigate the growing behavior and physico-mechanical properties of the resulting MBCs. The results of this study can provide valuable reference in MBC production and can be employed to enhance relevant strategies for the low-carbon disposal of SS.

## 2. Materials and Methods

### 2.1. Materials

**Figure 1:**
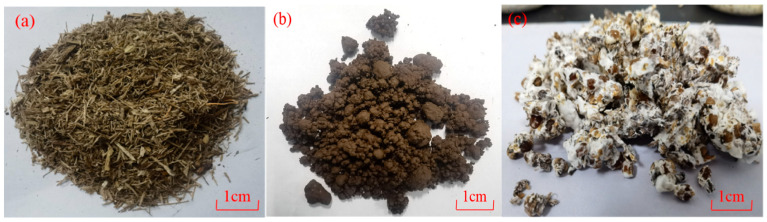
Pictures of substrate filler materials: (**a**) bagasse, (**b**) SS, (**c**) ready-made mycelium.

In this study, bagasse (Figure 1a) and SS (Figure 1b) were used as substrates. Bagasse was milled to different size ratios with a straw cutter and screening to exclude particles more than 10 mm (square-mesh screen of 9.5 mm). SS was acquired by anaerobic digestion and collected from Deyang Solid Waste Disposal Co., Ltd. (Deyang, China). The cellulose composition of the substrates is shown in Table 1 by the Van Soest method. Table 2 and Table 3 list the chemical compositions and other physical properties. The ready-made mycelium in this study, *Pleurotus ostreatus*, which consisted of 5% hyphal inoculum cultivated on the 95% wt. nutrient-rich wheat grains, was purchased from a commercial farm (Figure 1c).

### 2.2. Sample Preparation

#### 2.2.1. Preparation of Substrates

The bagasse was hand-mixed with enough deionized water to remove impurities. Then, this bagasse was autoclaved at 121 °C for 1 h (JSM280G-12, Ningbo Jiuxing Medical Equipment Co., Ltd., Ningbo, China) to sterilize it and then put into an ozone disinfection cabinet (ZTD50A-50, Guangzhou Wanbao Group Co., Ltd., Foshan, China) to avoid bacterial contamination until it cooled to room temperature. The same sterilization process was performed for the SS for 1.5 h twice to sterilize the various harmful components such as bacteria and viruses. To investigate the compatibility of fungi with SS, three different substrate types with bagasse as the control were designed to prepare MBCs with the ready-made mycelium, *Pleurotus ostreatus*, inoculation. Bagasse and SS were used alone and in combination at different mixture ratios, as shown in Table 4.

#### 2.2.2. Preparation of MBCs

**Figure 2:**
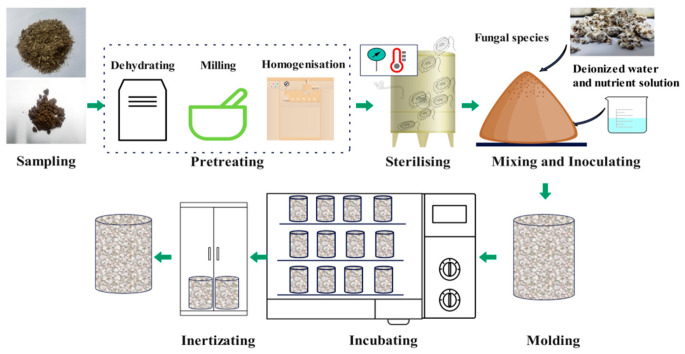
The flowchart showing the preparation of the MBCs.

The flowchart for preparing the MBCs is shown in Figure 2. The ready-made mycelium was removed from the polypropylene bottle and torn down to smaller particles. According to the formulation shown in Table 4, the mixture substrate was inoculated with the ready-made mycelium on a clean bench and was molded into a round-mouth cup with a diameter of 5 cm and a height of 8 cm without compaction. To avoid moisture evaporation, the top surface of the molds were sealed with plastic wrap. Moreover, holes were poked in the plastic wrap to ensure good ventilation. They were then placed in a curing box (SHBY-60b, Hebei Xinmeihua test Instrument Co., Ltd., Cangzhou, China) with humidity ranging from 60–70% and a temperature of 25 ± 1 °C. All the samples were watered with a mist spray every 24 h, and the growth state was recorded. A few days later, the MBCs were demolded and stored in a dark incubator at a temperature of 25 °C and 60% RH until they reached a constant mass to test the wet density, Then, they were oven-dried for 12 h at 80 °C for inertization, which was performed to deactivate the fungal organisms and test the dry density of the MBCs.

### 2.3. Testing and Characterization

#### 2.3.1. Appearance, Density, and Volume Shrinkage

The appearance of the MBCs at different ages were characterized by recording photos [31]. A greater number of hyphae indicated that the hyphae grew more rapidly at the same time and more nutrients were obtained from the substrates. This contrast of appearance was used to determine the biological compatibility of fungal species with SS.

The dry density, wet density, and volume shrinkage of the MBCs were calculated following Equations (1) and (2):

(1) $$\mathrm{Density}=\frac{m}{v}$$

(2) $$\mathrm{Volume}\;\mathrm{Shrinkage}=\frac{V_{2}-V_{1}}{V_{1}}$$

where m is the mass of the MBCs in g and v is the volume of the MBCs in cm^3^. V was calculated by the diameter and height. Three repeated tests were conducted for each sample and presented as the means ± standard deviation via Origin Pro 2021 (OriginLab, Northampton, MA, USA).

#### 2.3.2. Compressive Strength Test

The compression test was conducted via a microcomputer-controlled electronic universal testing machine (EHC-1300, Jinan Liangong testing Technology Co., Ltd., Jinan, China) with a constant rate of 0.5 kN/s. To reduce errors, three samples were tested, and the average value was taken as the final compressive strength of the MBCs.

#### 2.3.3. Thermal Conductivity Test

The thermal conductivity of the MBCs was determined using a thermal conductivity instrument (TPS2500S, Hot Disk Co., Ltd., Göteborg, Sweden) based on the ISO 22007-2 test standard [32]. Prior to testing, it was assured that the samples were completely and absolutely dry.

#### 2.3.4. FTIR Test

The FTIR spectra of the MBCs were recorded via a Spectrum One FTIR Spectrometer (Perkin Elmer, Waltham, MA, USA) in the range of 400–4000 cm^−1^.

#### 2.3.5. SEM Test

The morphology of all specimens was detected by a field emission scanning electron microscope (Zeiss Ultra 55, Zeiss Instrument Co., Ltd., Oberkochen, Germany). The separated cross-sections of the samples were cored into small pieces and used to observe internal surface structures. Before carrying out the tests, each sample was dried for 24 h at 80 °C to expedite fungal inertization then coated with a thin layer of gold for SEM analysis, and the surface morphology of the gold-coated samples was observed.

#### 2.3.6. TG Test

Thermogravimetric analysis was used to examine the thermal stability and weight loss of the samples using a thermoanalyzer (ZRT-B, Beijing Jingyi High-tech Instrument Co., Ltd., Beijing, China). The samples were heated under an air atmosphere from an ambient temperature to 800 °C at a heating rate of 5 °C per min.

## 3. Results and Discussion

### 3.1. Appearance

**Figure 3:**
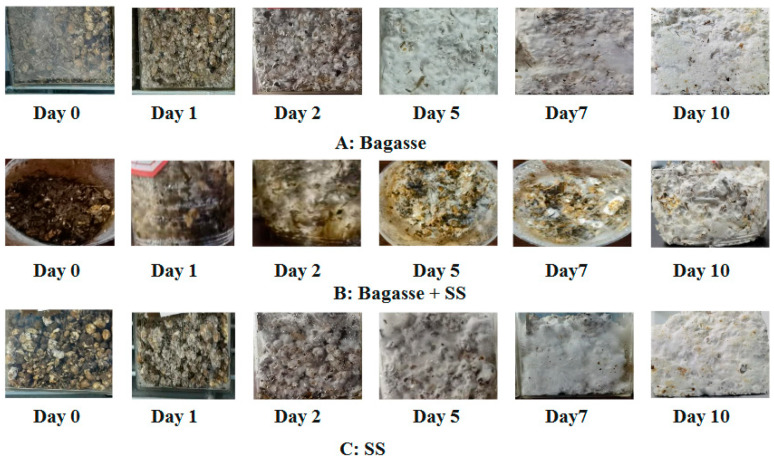
Growing process of MBCs derived from different substrates. (**A**): bagasse, (**B**): bagasse + SS, (**C**): SS.

Figure 3 shows the formation process of the MBCs. On day 1, some white hyphae were not visible to the naked eye because the fungal population was in a period of zero or low growth, referred to as the lag phase [33]. Over the next 24 h, the surfaces of the wheat grains were covered with a layer of white hyphae. The inoculated fungal cells grew accustomed to the new chemical and physical environment. Some fungal mycelia grew out of the substrate particles on day 5. In comparison, the sample cultured by bagasse showed that the hyphae grew more rapidly because the fungi degraded the substrates to obtain nutrients for vegetative growth by secreting cellulases, oxidases, phosphatases, chitinases, and proteases. The cellulases of *Pleurotus ostreatus* showed the natural affinity of hemicellulose [34]. As shown in Table 1, the bagasse contained more hemicellulose than the sewage sludge, and the fungi first consumed simple sugars (i.e., hemicellulose) rather than more complex sugars (i.e., cellulose). On day 10, all the samples were white on their external surfaces. Ready-made mycelia successfully colonized the substrates during the growth stage, resulting in signature white mycelial layers on the outer surface of the composites. The hyphae can enter the interspaces of substrates with the help of the expansion pressure of the growing hyphae; they then branch and extend into the interspaces and surfaces of filling materials to allow for cohesion in incoherent substrates [4,35] and confer three-dimensional interconnected networks to the resulting MBCs.

### 3.2. Density

**Figure 4:**
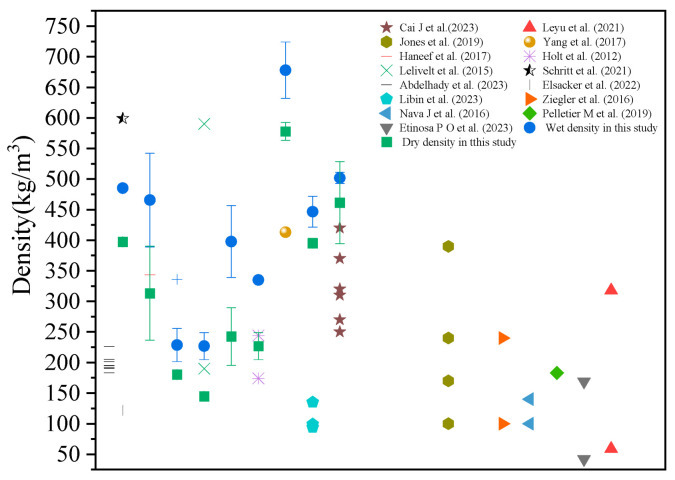
The comparison of density among the reference literature [4,7,10,13,18,24,36,37,38,39,40,41,42,43,44].

**Figure 5:**
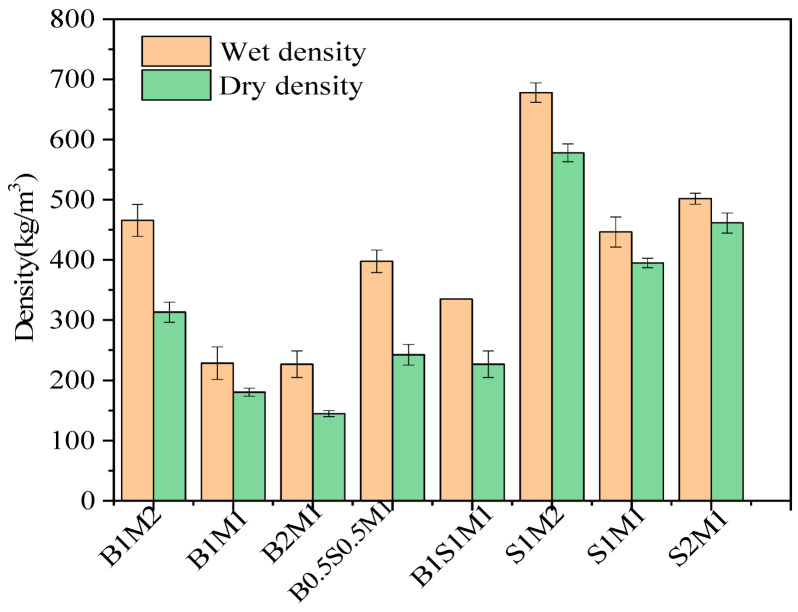
The density of the MBCs in Group 1, Group 2, and Group 3.

The data reported in the literature are compared in Figure 4. The density of the MBCs derived from different agricultural wastes or plant residues ranged from 100 to 700 kg/m^3^. Our data demonstrated that the density of the MBCs based on SS was acceptable for lightweight backfilling engineering and comparable to those prepared from agricultural wastes. The wet density and dry density of all samples are shown in Figure 5. Overall, the MBCs in Group 1 derived from bagasse as the sole substrate had relatively low densities. When the proportion of bagasse was increased from 1:2 to 2:1, the wet density and dry density of the MBCs decreased. When SS was added at different ratios in Group 2, the wet and dry densities of the MBCs increased. This was mainly attributed to the different densities of the substrates, as shown in Table 2. The heavy constituents in the substrate mixture subsequently increased, resulting in an increasing density of MBCs. The densities of the MBCs increased with the use of SS as the sole constituent. However, the histogram of Group 3 shows that the density curve with respect to the percentage of SS had an inflection point. Specifically, SS provided nutrients for vigorous mycelial growth, and the hyphae decomposed the substrates to reduce the density of the MBCs. When the ratio of SS to bagasse reached 1:1, the wet density and dry density were the lowest at 446 kg/m^3^ and 395 kg/m^3^, respectively.

### 3.3. Volume Shrinkage

**Figure 6:**
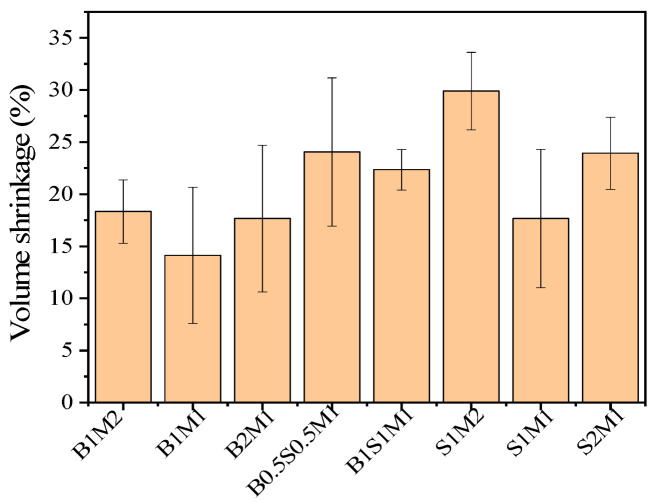
The histograms of volume shrinkage of MBCs.

The low volume shrinkage of the MBCs could contribute to the dimensional stability of the resulting product [10]. The volume shrinkage of the MBCs is shown in Figure 6. The volume shrinkage first decreased but then increased in each group. In comparison, the volume shrinkage of the S1M2 (29.90%) samples was greater than that of the other samples. The decreases in volume shrinkage of the B1S1M1 and S2M1 samples were 22.34% and 23.92%, respectively. The differences in volume shrinkage among B1M2, B1M1, B2M1, and S1M1 were very small. This phenomenon was attributed to residual water vaporization in the sterilized substrates and the high moisture content in the MBCs. After the samples were placed in an oven, most of the moisture was eliminated. As a result, the masses and volumes of the MBCs decreased. Furthermore, the standard deviation of the volume shrinkage for each MBC was greater than the standard deviation of the density. The available data on volume shrinkage were inconsistent, and the level of volume shrinkage was too high for dimensional stability in the product. The implication was that the prepared MBCs could be suitable only for applications where dimensional variability was permissible. Moreover, enhancing the anti-shrinkage potential of MBCs in subsequent investigations was considered necessary.

### 3.4. Compressive Strength

**Figure 7:**
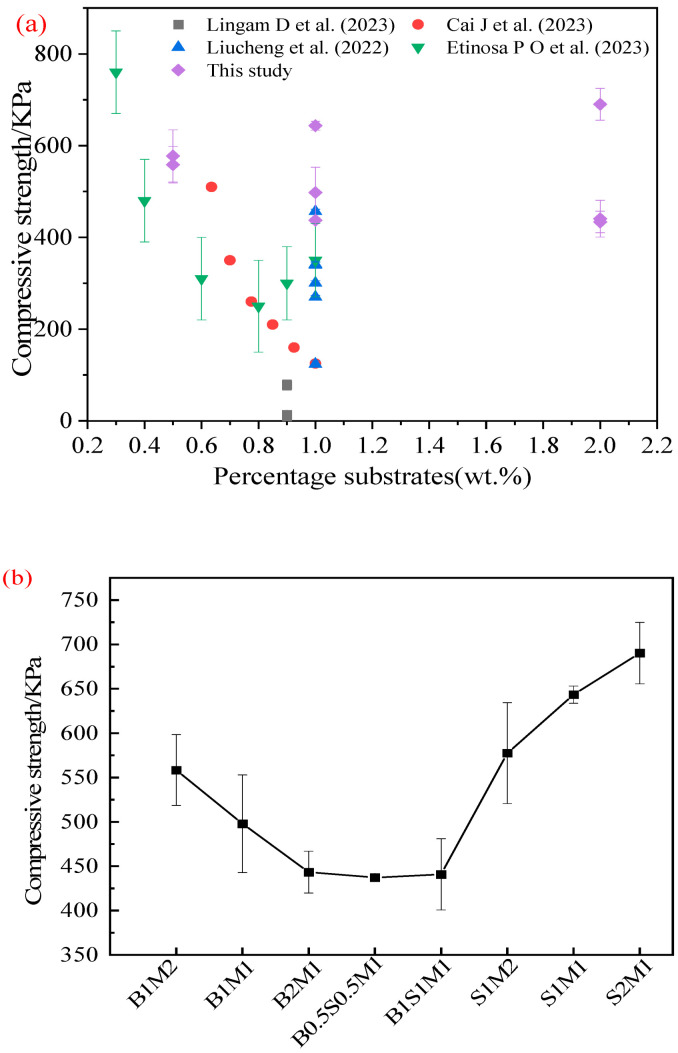
Compressive strengths of MBCs: (**a**) contrast of compressive strengths of MBCs reported in the literature; (**b**) compressive strengths in this work [13,31,44,45].

Figure 7a presents the compressive strengths of the samples reported in the published literature. The strength distributions of the MBCs with different substrates were wide and ranged from 7.7 to 760 KPa. As asserted in [27], while substrates with high cellulose content resulted in rapid fungal growth and high compressive strength, substrates with low nutrient contents could sustain fungal growth [39]. The compressive strength in this study is shown in Figure 7b. The compressive strength gradually decreased with the increasing bagasse–ready-made mycelium ratio, indicating that the strength of the material was dominated by the growth of ready-made mycelium. On the basis of density (Figure 5), the density clearly decreased, and strength decreased [4]. The compressive strength of the MBCs decreased with the increasing percentage of SS in the mixed substrate. However, the compressive strength in Group 3 showed the opposite trend when the substrate consisted of only SS. This finding further verified that the SS provided nutrients for mycelial growth on particulate-based lignocellulosic SS and that fungal cells assembled into filaments, forming a highly interlocked porous network structure binding the particulates of the SS together [18]. In addition, the organic components in the SS acted as nutrient sources, and inorganic matter played a role in the filling effect. The compressive strength of S1M2 was lower than that of S2M1 (Figure 7b). The filling and strengthening effects of the SS were most apparent with the increasing percentage of SS [44], which improved the mechanical properties of the final MBCs.

### 3.5. Thermal Conductivity

Table 5 shows that the thermal conductivity of B1M2 and S2M1 was 0.12 Wm^−1^K^−1^ and 0.13 Wm^−1^K^−1^, respectively. Under the same circumstances, the MBCs made from the SS had higher thermal conductivity than those constructed from the bagasse due to the greater amount of inorganic matter in the SS. By combining the dry density in Section 3.2, the thermal conductivity of B1M2 satisfied the standard range of T/CHTS 10165-2024 (Technical Guidelines for Application of Foamed Lightweight Soil in Highway, in which the limit λ of class W4 was ≤0.12 Wm^−1^K^−1^) [46]. The thermal conductivity of S2M1 was less than the class W5 standard (the limit λ of class W5 was ≤0.15 Wm^−1^K^−1^). The literature reported that as fungal degradation progressed, the thermal conductivity of the MBCs decreased gradually [47]. Therefore, the longer incubation time of the bagasse-based MBCs should be investigated in future studies.

### 3.6. The FT-IR Analysis

**Figure 8:**
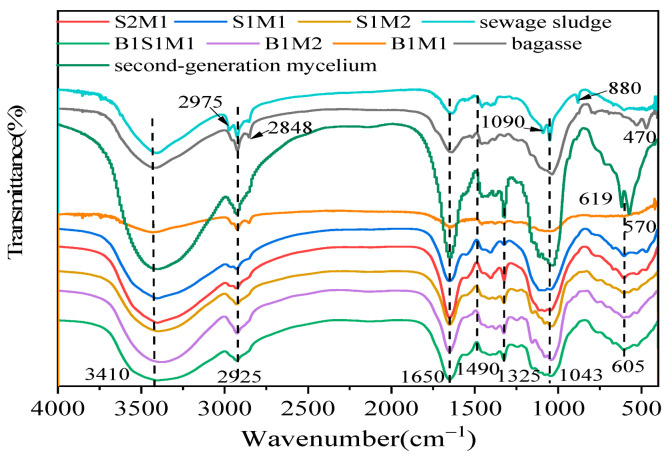
Fourier transform infrared (FT-IR) spectra of SS, bagasse, and representative MBCs.

**Figure 9:**
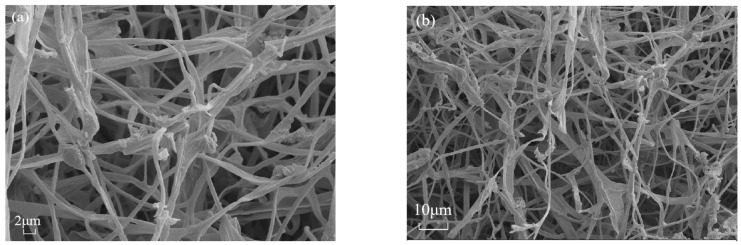

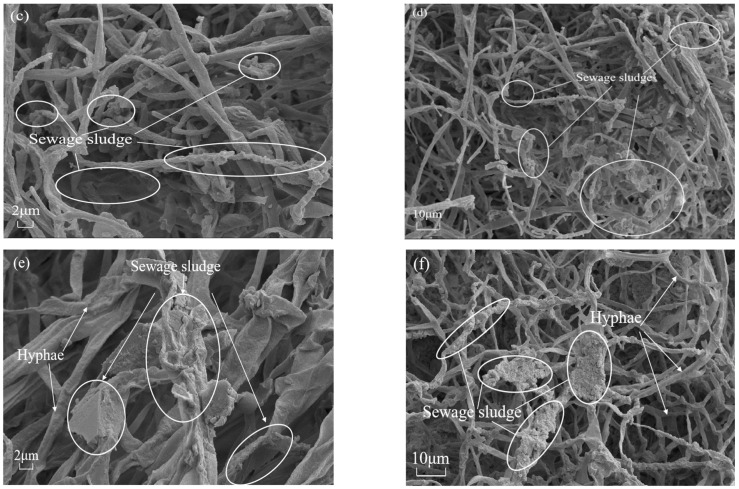
SEM images of MBCs with different substrates: (**a**,**b**) samples derived from bagasse alone; (**c**,**d**) samples derived from bagasse and SS; (**e**,**f**) samples derived from SS.

FT-IR spectroscopy was used to investigate the differences in the chemical compositions of the raw materials and the resulting MBCs. Figure 8 and Table 6 present the FT-IR spectra and corresponding peak assignments. The characteristics for the biomolecules listed in Table 1 and the elements listed in Table 2, such as lipids, proteins, and polysaccharides, were reflected in the FT-IR spectra. As shown in Figure 9 and Table 6, the bagasse presented signals typical of various functional groups, such as O-H stretching of polysaccharides (from cellulose and hemicellulose) at 3410 cm^−1^ [48] and CH symmetric and symmetric stretching at 2975 cm^−1^, 2925 cm^−1^, and 2848 cm^−1^ (waxes and oils) [49]. The signals for C=O stretching (called amide I) [50,51] and -NH_2_ at 1650 cm^−1^ and CH_2_ bending at 1490 cm^−1^ are related to proteins. The peak at 1325 cm^−1^ was attributed to the NH_2_ stretching vibration in amines (amide III) [17]. The C-C stretching vibration at 1043 cm^−1^ was attributed to proteins, lignin, and polysaccharides. Compared with those in bagasse, the signals of proteins and polysaccharides detected in the SS and ready-made mycelium were greater. For example, the SS showed a characteristic peak for the stretching vibration of C-O groups from polysaccharides at 1090 cm^−1^ and the glucan β-anomer C-H bending stretching vibration of cellulose at 880 cm^−1^ [17,48,52]. Additionally, the ready-made mycelium presented stronger characteristic peaks at 1650 cm^−1^, 1325 cm^−1^, 1050 cm^−1^, and 605 cm^−1^. The MBCs presented characteristic bands common to the starting materials (bagasse and SS) combined with absorption bands from the ready-made mycelium. These findings were similar to those reported in the literature, with the bands identified in the fungal species studied aligning with the main bands reported previously. Remarkably, the peaks for amide I at 1650 cm^−1^, amide III at 1325 cm^−1^, and C-H below 800 cm^−1^ were weaker in the MBCs than in the raw materials. The chemical characteristics of the starting materials were clearly responsible for the distinct changes in the infrared spectra of the MBCs [12,17,25,31,48]. Bands related to proteins, cellulose, and polysaccharides (1090 cm^−1^, 880 cm^−1^, 619 cm^−1^) were not detected in any of the MBCs, indicating the decomposition of proteins, cellulose, and hemicellulose. The enzymes of fungi secreted during *Pleurotus ostreatus* growth facilitated the rapid decomposition of the SS and bagasse through the digestion of proteins, cellulose, and hemicellulose.

### 3.7. SEM Analysis

Figure 9 presents the images of the representative MBCs. Two SEM images per sample were captured at 1000× and 2000× magnification levels. The SEM images confirmed that the interwoven network of the MBCs was generated by filamentous hyphae and aggregated fibrous mycelia on the bagasse and SS. The resulting bagasse-based MBCs were relatively thin. There were more air voids between the bagasse fiber in the interwoven hyphal networks (Figure 9a,b). The addition of SS led to tenacious and dense hyphal growth, as shown in Figure 9c,d. The SEM images (Figure 9e,f) for S1M1 revealed highly clustered SS and hyphae that were relatively strong and thick. Because the presence of proteins, carbohydrates, and lipids in the SS facilitated hyphal growth and nutrient utilization [25], the exterior surfaces of the hyphae overlapped with those of the SS layers, and the voids were filled with SS, which resulted in a relatively dense network of mycelial filaments, as shown in Figure 9e,f, to further verify the filling and strengthening effects of SS.

### 3.8. TGA Analysis

**Figure 10:**
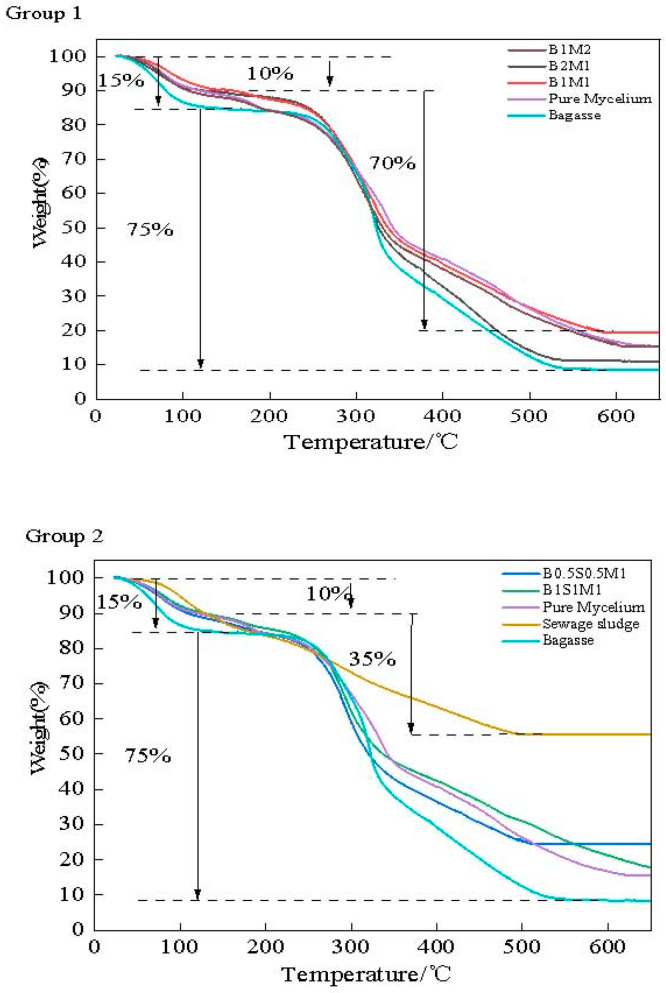

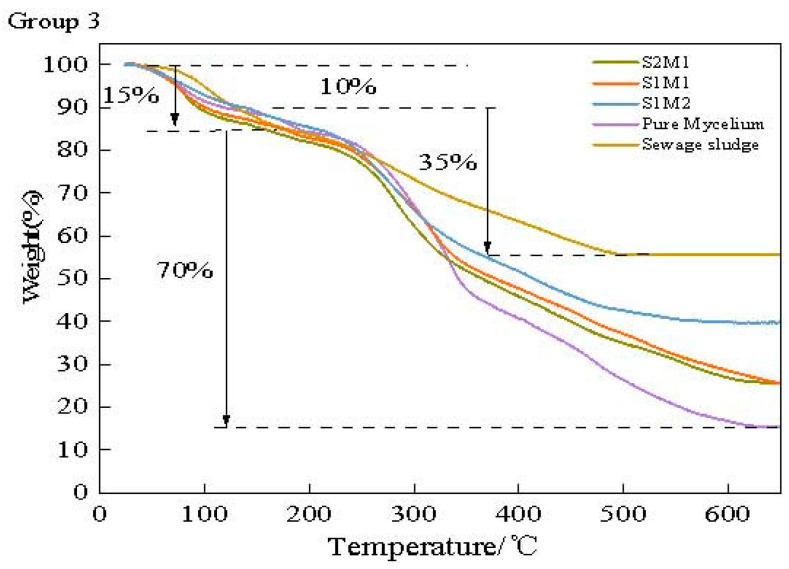
The TGA analysis of MBCs from 30–600 °C.

The thermal stability of the MBCs was analyzed using thermogravimetric analysis (TGA), as shown in Figure 10. Two decomposition stages were observed in all treatments. At 80–100 °C, 10–15% of the weight loss was attributed to the evaporation front for free water and chemically coupled water. During the second stage, distinct degradation occurred between 250 °C and 500 °C. Approximately 70–75% of the weight was lost, as shown in Figure 10 (Group 1), whereas approximately 60% of the weight was lost, as shown in Group 3. Owing to the presence of inorganic matter in the SS, which may greatly increase thermal stability, the final residue of the SS at 500 °C was quite high, at 55.67%, whereas the residue of the bagasse was only 12.44%. However, thermal degradation of the MBCs began at 250 °C and constituted more than half of the mass loss at 350 °C. The loss of mass remained constant at 500 °C. These values were within the degradation ranges of bagasse and SS. The cellulose and hemicellulose in the fibrous mycelium broke down at 200–300 °C, whereas lignin decomposed at a temperature of approximately 400 °C [53]. Therefore, it was deduced that the thermal degradation of the cellulose, hemicellulose, and lignin in the MBCs was the cause of the rapid decrease in the second decomposition stage of all the MBCs. On the basis of the thermal stability observed via TGA, the samples in Group 3 were determined to be more stable. Hence, the addition of SS to the MBCs resulted in improved thermal stability and resistance to thermal degradation.

## 4. Conclusions

In this study, the potential for producing MBCs using SS was explored. The MBCs were prepared by *Pleurotus ostreatus* cultured on SS and bagasse as substrates. The physico-mechanical, morphological, and thermal stability properties of the MBCs were studied. On the basis of a series of experimental tests, the following conclusions were drawn:

(1) The results proved that the ready-made mycelium grown on the SS substrate bound together to form a mycelial network on day 10. The fungi had good biological compatibility with the SS. The density of the resulting MBCs increased with increasing SS content.
(2) Owing to the filling effect and strengthening effect of SS, the compressive strengths of the MBCs increased significantly with increasing SS proportion. The compressive strength of the MBC prepared with an SS–ready-made mycelium ratio of 2:1 reached 690.20 KPa.
(3) The thermal conductivity of the MBCs was comparable to those of the foamed lightweight soil used in highways. However, the mycelium as a natural binder brings significantly better environmental benefits than EPS and cement, which can cause great damage to the environment due to their high energy consumption and carbon emissions during the production process.
(4) The microstructure revealed that the SS supported more robust growth of hyphae and resulted in stronger MBCs than the bagasse. Adding SS improved the thermal stability and thermal degradation resistance of the MBCs.

This is the first study reporting the use of SS to manufacture MBCs, and it highlights the potential use of SS to produce a range of MBCs. Therefore, the mechanism of SS promoting mycelial growth must still be elucidated, and accurate biological tests are needed to further characterize the digestion process of mycelia for the carbon and nitrogen sources present in SS. Further studies are recommended to optimize the manufacturing procedures to improve their performance and address the technical barriers for their application.

## Figures and Tables

**Table 1:** Cellulose composition of the substrates.

Types	Cellulose (%)	Hemicellulose (%)	Lignin (%)
Bagasse	21.3	36.1	41.1
SS	64.4	2.7	30.4

**Table 2:** Element composition and physical properties of the substrates.

Types	N (%)	C (%)	H (%)	S (%)	C/N	Dry Density (kg/m^3^)	Wet Density (kg/m^3^)	Moisture Content (wt%)
Bagasse	0.32	44.96	6.89	0.03	138.33	86.65 ± 7.09	317.12 ± 15.08	87.56 ± 1.74
SS	2.94	15.03	4.90	3.18	5.11	670.68 ± 19.29	547.90 ± 23.23	68.91 ± 0.15

Note: The moisture content of the substrates was tested after sterilization.

**Table 3:** Chemical composition of the substrates.

Types (%)	SiO_2_	Al_2_O_3_	Fe_2_O_3_	CaO	Loss
Bagasse	6.33	0.32	0.12	0.36	92.41
SS	16.69	10.92	0.93	23.16	43.38

**Table 4:** Formulation of MBCs derived from different substrates.

Group	The Protocols of Proportion of MBCs	Ratio (Mass)	Label
Group 1	Bagasse–ready-made mycelium	1:2	B1M2
		1:1	B1M1
		2:1	B2M1
Group 2	Bagasse–SS–ready-made mycelium	0.5:0.5:1	B0.5S0.5M1
		1:1:1	B1S1M1
Group 3	SS–ready-made mycelium	1:2	S1M2
		1:1	S1M1
		2:1	S2M1

Note: Group 1 samples were fed solely on bagasse as the control. In Group 2, bagasse and ready-made mycelium were mixed with SS for further exploration. Additionally, experimental exploration in Group 3 was performed to estimate whether SS as mono-component could offer the supplements for ready-made mycelium.

**Table 5:** Thermal conductivity of the MBCs.

Label	Temperature/°C	Thermal Conductivity (Wm^−1^K^−1^)
B1M2	25	0.12 ± 2.52 × 10^−4^
S2M1	25	0.13 ± 1.15 × 10^−4^

**Table 6:** FT-IR spectra and the assignment of peaks in Figure 8.

IR Shift (cm^−1^)	Peak Detected (cm^−1^)	Assignment	Main Contribution
3600–3300	3410	O-H s	Cellulose and hemicellulose
3050–2800	2975, 2925, 2848	CH s, as	Lipids, waxes, and oils
1700–1600	1650	C=O (Amide I), -NH_2_	Proteins
1500–1400	1490	C=O s	Proteins
1400–1200	1325	Amide III	Lipids and lignin
1200–1000	1090, 1043	C-C s	Proteins, lignin, and polysaccharides
1000–800	880	C-H b	Cellulose
Below 800	619, 605, 570, 470	C-H b	Polysaccharides

Note: The letters s, as, and b denote the symmetric, asymmetric, and bending stretching vibration, respectively.

## Data Availability

The original contributions presented in this study are included in the article. Further inquiries can be directed to the corresponding author.

## Abbreviations

The following abbreviations are used in this manuscript:

MBCs	mycelium-based composites
SS	sewage sludge
LBMs	lightweight backfill materials
EPS	expanded polystyrene
